# Early Aortic Valve Replacement of Asymptomatic Severe Aortic Stenosis: A Meta‐Analysis of Randomized Controlled Trials

**DOI:** 10.1161/JAHA.125.041283

**Published:** 2025-08-20

**Authors:** Qingchun Song, Ruilin Liu, Kai Yang, Xiaokang Tu, Haoyu Tan, Chengming Fan, Xiaoxiao Li

**Affiliations:** ^1^ Department of Cardiovascular Surgery, The Second Xiangya Hospital Central South University Changsha Hunan China; ^2^ Department of Plastic and Aesthetic(Burn)Surgery, Second Xiangya Hospital Central South University Changsha China; ^3^ Hunan Key Laboratory of Joint Degeneration and Injury, Xiangya Hospital Central South University Changsha China

**Keywords:** aortic valve replacement, asymptomatic, meta‐analysis, severe aortic stenosis, Aortic Valve Replacement/Transcather Aortic Valve Implantation

## Abstract

**Background:**

Asymptomatic severe aortic stenosis may lead to the progression to both symptoms and adverse outcomes if left untreated. The appropriate timing of intervention for these patients remains controversial, and any decision requires careful assessment.

**Methods:**

We searched PubMed, Web of Science, EMBASE, and Cochrane Library for randomized controlled trials comparing early aortic valve replacement with conservative management in asymptomatic patients with severe aortic stenosis until December 2024. The primary efficacy outcome was a composite of all‐cause mortality, hospitalization for cardiovascular causes, stroke, and myocardial infarction. We expressed outcome data as risk ratios (RRs) with 95% CIs.

**Results:**

We included 4 randomized controlled trials involving 1427 patients. Early aortic valve replacement significantly reduced the incidence of the composite outcome when compared with conservative management (29.2% versus 53.7%; RR, 0.56 [95% CI, 0.49–0.64]; I^2^=60%). Significant reductions were also detected in all‐cause mortality (10.0% versus 13.7%; RR, 0.74 [95% CI, 0.55–0.99]; I^2^=58%), hospitalization for cardiovascular causes (14.6% versus 32.5%; RR, 0.48 [95% CI, 0.39–0.58]; I^2^=26%), and stroke (4.5% versus 7.2% RR, 0.62 [95% CI, 0.40–0.95]; I^2^=0%). No significant difference was observed in cardiac‐specific mortality (8.3% versus 16% RR, 0.68 [95% CI, 0.40–1.16]; I^2^=65%) and myocardial infarction (0.6% versus 4.6%; RR, 0.21 [95% CI, 0.04–1.19]; I^2^=0%).

**Conclusions:**

Our meta‐analysis of randomized controlled trials showed that among patients with asymptomatic severe aortic stenosis, a strategy of early aortic valve interventions was superior to conservative treatments in reducing the primary composite outcome of all‐cause mortality, hospitalization for cardiovascular causes, stroke, and myocardial infarction. However, significant differences in the rates of cardiac‐specific mortality were not apparent.

**Nonstandard Abbreviations and Acronyms**
AS: aortic stenosis
AVR: aortic valve replacement
CM: conservative management
RCT: randomized controlled trial
RECOVERY: Randomized Comparison of Early Surgery versus Conventional Treatment in Very Severe Aortic Stenosis
SAVR: surgical aortic valve replacement
TAVR: transcatheter aortic valve replacement

**Clinical Perspective**

**What Is New?**

- This systematic review and meta‐analysis of 4 studies including 1427 asymptomatic patients with severe aortic stenosis revealed that early aortic valve replacement could significantly reducing the primary composite outcome of all‐cause mortality, hospitalization for cardiovascular causes, stroke, and myocardial infarction compared with conservative management for asymptomatic severe aortic stenosis.

**What Are the Clinical Implications?**

- Early intervention should be considered in asymptomatic patients with severe aortic stenosis to prevent the onset of symptoms and reduce the risk of unplanned hospitalizations. It is essential to take into account not only the degree of aortic valve stenosis but also patient‐specific factors, including age, the presence of comorbidities, and overall clinical status in shared decision‐making.

Aortic stenosis (AS) is the one of the most common forms of valvular heart disease, affecting >3% of adults in developed countries.^1^ Between one‐third and one‐half of patients with severe AS are asymptomatic at diagnosis.^2^ Previous perspective only supports the relationship between AS symptom onset and sharply decreased survival in untreated disease. However, more recent data have emerged to demonstrate that asymptomatic severe AS may lead to the progression to both symptoms and adverse outcomes if left untreated.^3^, ^4^, ^5^

According to the 2020 American College of Cardiology/American Heart Association guideline for managing valvular heart disease, aortic valve intervention is generally recommended for patients with severe AS presenting with symptoms or signs of ventricular decompensation.^6^ For asymptomatic AS, guidelines based on expert opinion and nonrandomized data recommend watchful waiting, with aortic valve intervention deferred until symptom onset or a left ventricular ejection fraction <50%. Nevertheless, the appropriate timing of intervention for these patients remains controversial, and any decision requires careful assessment.^7^

Several meta‐analyses have demonstrated the benefits of early intervention in patients with asymptomatic severe AS.^8^, ^9^, ^10^ However, the majority of included studies were observational or enrolled small numbers of relatively young and low‐risk patients.^11^, ^12^, ^13^, ^14^, ^15^, ^16^, ^17^ The primary aim of this study is to comprehensively review the available evidence from prospective randomized controlled trials (RCTs) to determine the optimal management strategy for patients with asymptomatic severe AS.

## METHODS

The authors declare that all supporting data are available within the article and its online supplementary files. This analysis was performed following the registered protocol in the International Prospective Register of Systematic Reviews (PROSPERO) (registration number: CRD42024623710). The methodology and study selection are reported in compliance with the Preferred Reporting Items for Systematic Reviews and Meta‐Analyses (PRISMA) guidelines.^18^

This study is not human subjects research and was judged exempt from institutional review board review.

### Data Sources and Search Strategy

A systematic literature search was performed in 4 databases: PubMed, Web of Science, EMBASE, and Cochrane Library, to identify articles published up to November 2024, with no restrictions on publication year or language. The search strategy in our analysis was designed based on the PICOS framework, focusing on 2 key components: asymptomatic patients with severe AS and early interventions including surgical aortic valve replacement (SAVR) and transcatheter aortic valve replacement (TAVR). Details of the specific search strategy are documented in Table S1.

### Study Selection

This study aimed to include RCTs assessing the benefits of early aortic valve replacement (AVR) compared with conservative management (CM) in asymptomatic patients with severe AS. Studies meeting the following criteria were included: (1) adult patients older than 18 years; (2) prospective randomized clinical trials; and (3) availability of data on all‐cause mortality, cardiac‐specific mortality, heart failure hospitalization, stroke, or myocardial infarction. Exclusion criteria were as follows: (1) patient age under 18 years; (2) studies without randomization; and (3) trials without control arm.

Two researchers (QCS and RLL) independently reviewed the titles and abstracts of retrieved articles using NoteExpress v3.9.0 (AegeanSoftware Corp, Beijing, China) and subsequently assessed the full texts to identify studies meeting the inclusion criteria. Discrepancies between researchers were resolved by mutual consensus with a third researcher (HYT).

### Data Extraction and Quality Appraisal

Two researchers (QCS and KY) independently extracted data from selected studies using Microsoft Excel 2017 (Microsoft, Redmond, WA) and adhered to a structured data extraction form. The form included study characteristics (publication year, study design, study region, patient population, degree of aortic stenosis, follow‐up duration, and primary and secondary outcomes), baseline characteristics (mean age, sex, body surface area, body mass index, hypertension, diabetes, smoking history, hyperlipidemia, coronary artery disease, echocardiographic findings), intervention (early SAVR or TAVR), and outcome measures (all‐cause mortality, cardiac‐specific mortality, heart failure hospitalization, stroke, or myocardial infarction). Two researchers (QCS and RLL) evaluated the quality of each included trial using the Cochrane Risk of Bias Tool 1 (ROB1).^19^ We resolved discrepancies by mutual consensus with a third researcher (HYT).

### Clinical Outcomes

The primary end point was a composite outcome of all‐cause mortality, hospitalization for cardiovascular causes, and major cardiovascular events including stroke and myocardial infarction. Secondary outcomes included the individual components of the composite primary end point and cardiac‐specific mortality for both groups.

### Statistical Analysis

A pairwise meta‐analysis was performed to compare early AVR with CM in asymptomatic patients with severe AS. The risk ratio (RR) and their corresponding 95% CIs were used as the primary effect estimate for synthesizing dichotomous outcome data. Given the small number of included studies (n=4), and the potential limitations of random‐effects models in such settings, we used a fixed‐effect model to estimate the pooled effect size.^20^, ^21^ Heterogeneity of the effect size across the studies was assessed using the I^2^ statistic (I^2^ <25% representing low heterogeneity, 25% to 50% moderate, and I^2^ >50% high) and the Q statistic (*P* <0.1 indicating heterogeneity). Although our main meta‐analysis adopted a fixed‐effect model due to the small number of included studies and concern over unstable heterogeneity estimates, we calculated a prediction interval (PI) based on a random‐effects model using the Restricted Maximum Likelihood method to estimate between‐study variance. This PI was provided as a sensitivity analysis. Additionally, we performed univariate meta‐regression using a random‐effects model (Restricted Maximum Likelihood method) to explore whether the observed heterogeneity could be partially explained by study‐level covariates.^21^ The meta‐regression analysis was conducted basing on predefined moderator variables including age, body mass index, peak aortic jet velocity, and follow‐up duration. A leave‐one‐out sensitivity analysis was also conducted, excluding 1 study at a time and repeating the analysis. Additionally, considering the differential risk profiles of TAVR versus SAVR patients, a subgroup analysis basing on different intervention strategies was conducted. Publication bias was evaluated using funnel plots. Grading of Recommendations Assessment, Development and Evaluation methodology was used to assess the certainty of the overall evidence for the results in our analysis. The quality of evidence ranged from very low certainty to high certainty. GRADEpro software (McMaster University, Ontario, Canada) was used to draft the Grading of Recommendations Assessment, Development and Evaluation tables (Table S2).

Meta‐analyses in our analysis were conducted using Review Manager 5.3 software (Cochrane Collaboration, Copenhagen, Denmark), The “metafor” package were used to perform power analysis of the meta‐analysis in the R4.3.3 software (R Foundation for Statistical Computing, Vienna, Austria) and statistically significant results were defined as 2‐sided *P* values of <0.05.

### Results

**Figure 1:**
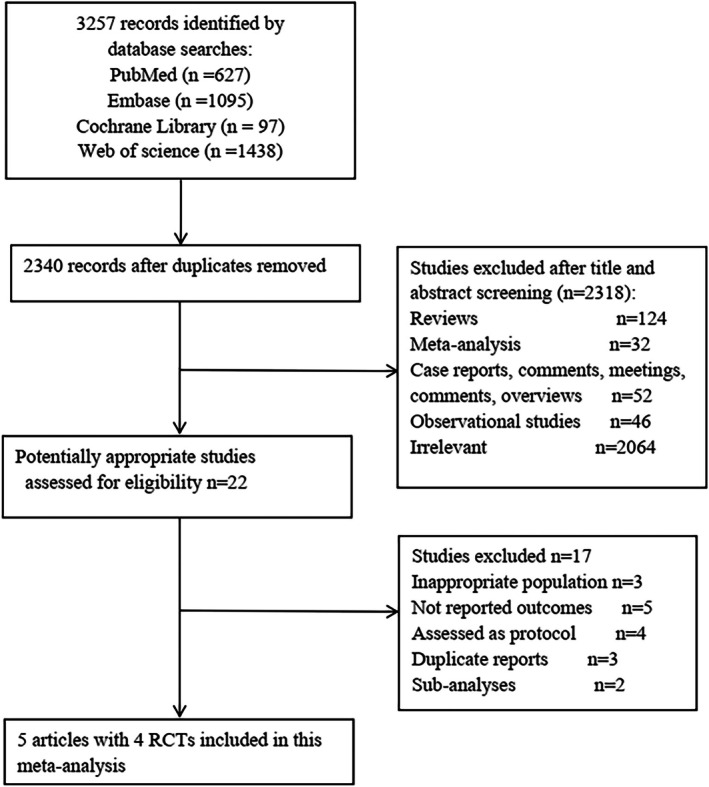
Selection process of included studies. RCTs indicates randomized controlled trials.

A total of 3257 articles were identified in the literature search, with 2340 remaining after duplicate removal. After thorough screening of titles, abstracts, and full texts, 5 articles comprising 4 RCTs were ultimately included in the analysis (Figure 1).^16^, ^17^, ^22^, ^23^

### Characteristics of the Eligible Trials

All the trials were conducted in developed countries including South Korea, the United States, Australia, and European countries. The mean follow‐up duration ranged from 42 months in the EVOLVED (Early Valve Replacement Guided by Biomarkers of Left Ventricular Decompensation in Asymptomatic Patients with Severe Aortic Stenosis) trial to 74 months in the RECOVERY (Randomized Comparison of Early Surgery versus Conventional Treatment in Very Severe Aortic Stenosis) trial. The participants in the RECOVERY trial had very severe AS, while in other trials they had severe AS. All the primary outcomes of the included trials were composite outcomes. The main study characteristics are shown in Table 1.

**Table 1:** Characteristics of the Included Studies

Trials	Study design	Country	Patient population	Degree of aortic stenosis	Follow‐up duration	Primary outcome	Secondary outcome
RECOVERY, 2020	RCT	Korea	20 to 80 y	Aortic‐valve area of 0.75 cm^2^ or less with either a peak aortic jet velocity of 4.5 m/s or greater or a mean transaortic gradient of 50 mm Hg or greater	6.2 y (5.0–7.4)	A composite of operative mortality or death from cardiovascular causes during the follow‐up period	Death from any cause, repeat aortic‐valve surgery, clinical thromboembolic events, and hospitalization for HF during follow‐up
AVATAR Trial, 2021	RCT	European countries	>18 y	Valve area ≤1 cm^2^ with aortic jet velocity >4 m/s or a mean transaortic gradient ≥40 mm Hg	60 mo	A composite of all‐cause mortality or major adverse cardiovascular events and unplanned HF hospitalization	In‐hospital and 30‐day postoperative mortality, repeat aortic valve surgery in operated patients in both groups
EVOLVED, 2024	RCT	UK and Australia	≥18 y	Aortic valve peak velocity ≥4.0 m/s or an aortic valve peak velocity ≥3.5 m/s with an indexed aortic valve area <0.6 cm^2^/m^2^	42 mo	A composite of all‐cause mortality or unplanned aortic stenosis–related hospitalization during the follow‐up period	Cardiovascular death; aortic stenosis–related death; sudden cardiac death; stroke; endocarditis; implantation of a cardiac pacemaker, resynchronization device, or automated cardioverter defibrillator; and postoperative complications
EARLY TAVR, 2024	RCT	The United States and Canada	≥65 y	AVA ≤1 cm^2^ or AVA index ≤0.6 cm^2^/m^2^ and Peak jet velocity ≥4 m/s or mean gradient ≥40 mm Hg	3.8 y (2.8– 5.0)	A composite of death from any cause, stroke, or unplanned hospitalization for cardiovascular causes	Favorable outcome, integrated measures of LV and LA health, change in LV ejection fraction, new‐onset atrial fibrillation, death or disabling stroke

AVA indicates aortic valve area; HF, heart failure; LA, left atrium; LV, left ventricle; and RCT, randomized controlled trials.

A total of 1427 participants were included across the 4 trials, with 719 receiving early AVR and 708 undergoing CM. The mean age of participants ranged from 63 to 76 years. The majority of participants were male, with the proportion of males ranging from 41% to 73% across the included studies. The severity of AS was assessed using cardiac Doppler echocardiography in all studies. The mean aortic valve area ranged from 0.6 to 0.8 cm^2^, with mean transaortic pressure gradient ranging from 45 to 64 mm Hg. The mean left ventricular ejection fraction exceeded 60%. Exercise testing was performed in all candidates in the AVATAR (Aortic Valve Replacement Versus Conservative Treatment in Asymptomatic Severe Aortic Stenosis) trial, and selectively performed in other trials. The baseline characteristics are presented in Table 2.

**Table 2:** Baseline Characteristics of Included Trials

Variable (Early AVR vs conservative management)	RECOVERY, 2020	AVATAR, 2021	EVOLVED, 2024	EARLY TAVR, 2024
Age, mean, y	65.0±7.8 vs 63.4±10.7	68 (63–73) vs 69 (64–74.5)	75 (68–79) vs 76 (68–80)	76.0±6.0 vs 75.6±6.0
Male, n, %	37 (51) vs 34 (47)	32 (59.0) vs 35 (55.7)	82 (73) vs 79 (71)	324 (71.2) vs 299 (67)
Patients randomized, n	73 vs 72	78 vs 79	113 vs 111	455 vs 446
Body‐surface area, m^2^	1.69±0.17 vs 1.64±0.17	1.9 (1.8–2.1) vs 1.9 (1.8–2.0)	NA	NA
Body mass index, kg/m^2^	24.7±3.4 vs 24.0±2.6	27.2 (25.6–29.3) vs 27.4 (25.4–30.9)	27.2 (24.4–31.1) vs 27.8 (24.8–31.1)	28.4±4.6 vs 28.6±4.8
Hypertension, n, %	40 (55) vs 39 (54)	69 (88.4) vs 70 (88.6)	76 (67) vs 70 (63)	369 (81.1) vs 365 (81.8)
Diabetes, n, (%)	13 (18) vs 7 (10)	14 (17.9) vs 23 (29.1)	15 (13) vs 26 (23)	119 (26.2) 114 (25.6)
History of smoking, n, (%)	19 (26) vs 21 (29)	16 (20.5) vs 14 (17.7)	51 (45) vs 55 (50)	NA
Hyperlipidemia, n, (%)	41 (56) vs 42 (58)	31 (39.7) vs 28 (35.4)	55 (49) vs 56 (50)	375 (82.4) vs 347 (77.8)
Coronary artery disease, n, (%)	1 (2) vs 5 (7)	1 (1.3) vs 3 (3.8)	NA	133 (29.2) vs 113 (25.3)
Echocardiographic findings				
Peak aortic jet velocity, m/s	5.14±0.52 vs 5.04±0.44	4.5 (4.3–4.8) vs 4.5 (4.2–4.7)	4.3±0.5 vs 4.4±0.5	4.3±0.5 vs 4.4±0.4
Mean transaortic pressure gradient, mm Hg	64.3±14.4 vs 62.7±12.4	50.7 (45–58) vs 49.5 (43–58)	45.2±11.5 vs 45.0±10.2	46.5±10.1 47.3±10.6
Valve area, cm^2^	0.63±0.09 vs 0.64±0.09	0.73 (0.5–0.8) vs 0.74 (0.6–0.9)	0.8±0.2 vs 0.8±0.2	0.8±0.2 vs 0.8±0.2
Valve area index, cm^2^/m^2^	0.38±0.06 vs 0.39±0.07	0.37 (0.3–0.4) vs 0.37 (0.3–0.4)	NA	NA
LVMI, g/m^2^	135.6±38.2 vs 133.7±31.1	152 (133.1–173.5) vs 160 (139–180.8)	NA	NA
LVEF, %	64.8±5.2 vs 64.8±4.1	70 (65–76) vs 69 (63–75)	NA	67.4±6.5 vs 67.4±6.7

AVR indicates aortic valve replacement; LVEF, left ventricular ejection fraction; LVMI, left ventricular mass index; and NA, not applicable.

The assessment of bias risk in each study is presented in Figure S1. The random sequence generation method was not reported in the EVOLVED trial, which was judged as unclear risk of bias. Blinding of participants and study personnel was not feasible in any of the trials due to the overt nature of the interventions.

### Primary Outcome

**Figure 2:**
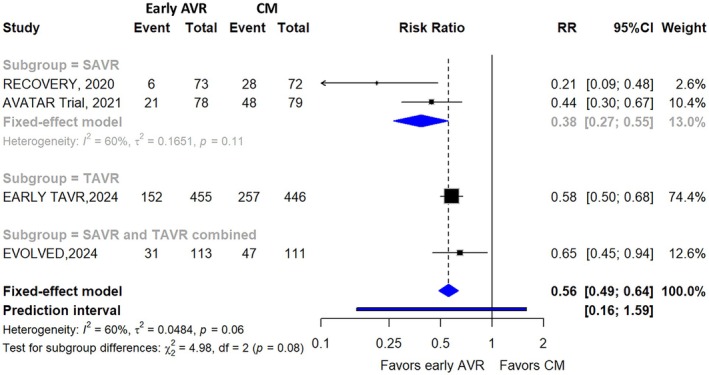
Forest plot illustrating the association of early AVR and conservative management with the primary composite outcome. AVR indicates aortic valve replacement; CM, conservative management; RR, risk ratio; SAVR, surgical aortic valve replacement; and TAVR, transcatheter aortic valve replacement.

The primary outcome was a composite outcome of all‐cause mortality, hospitalization for cardiovascular causes, stroke, and myocardial infarction. Pooled results showed that early AVR significantly reduced the incidence of the composite outcome when compared with CM (29.2% versus 53.7%; RR, 0.56 [95% CI, 0.49–0.64]; I^2^=60%) (Figure 2). The certainty of evidence was deemed low (Table S2). While the fixed‐effect model was used as the primary analytical approach due to the small number of included studies, we acknowledge that the observed moderate heterogeneity (I^2^=60%) warrants a deeper investigation into between‐study variability. To address this, we calculated a 95% PI using a random‐effects model as a sensitivity analysis. The resulting PI ranged from 0.18 to 1.59, indicating substantial potential variation in the true effect size across future or similar settings. The leave‐one‐out sensitivity analysis showed no significant alteration in the effect estimate. Of note, after excluding the RECOVERY, no statistical evidence of heterogeneity was detected in the pooled outcome with I^2^=0% (Table S3). Subgroup analysis of studies with different intervention strategies did not reveal any substantial change (Figure 2). The funnel plots did not reveal obvious publication bias for the outcomes. (Figure S2).

Meta‐regression analysis revealed that mean age and peak aortic jet velocity were significantly associated with the effect size. Specifically, for each 1‐year increase in mean age across studies, the log risk ratio (log RR) increased by 0.053 (β=0.053; *P*=0.0214), suggesting a diminished treatment effect in older populations. Conversely, for each 1 m/s increase in peak aortic jet velocity, the log RR decreased by 1.121 (β=−1.121; *P*=0.0416), indicating a stronger treatment effect in patients with higher baseline. These findings suggest that part of the observed heterogeneity may be attributable to differences in baseline age and severity of aortic stenosis across studies (Table S4).

### Secondary Outcomes

**Figure 3:**
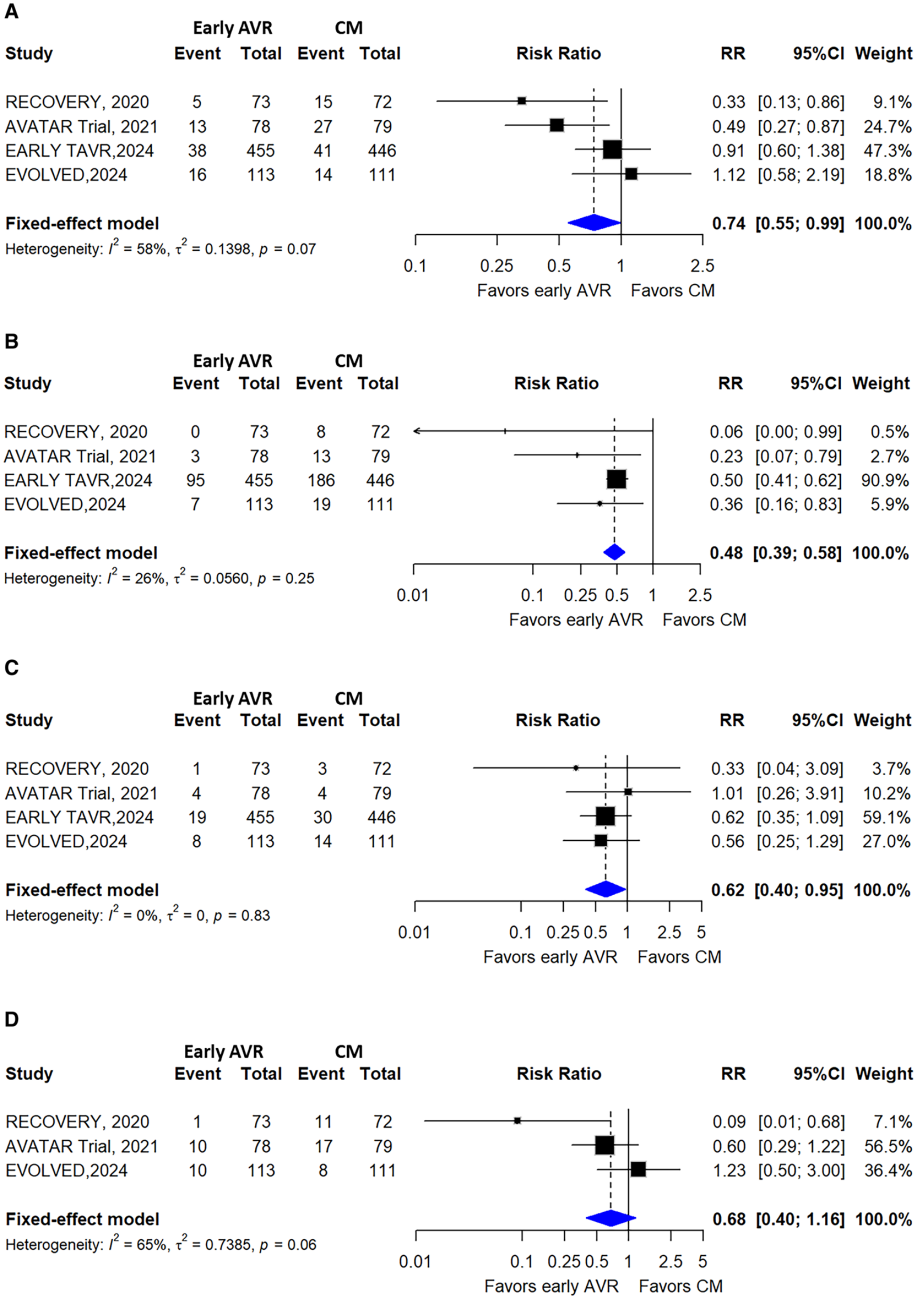
Forest plot illustrating the association of early AVR and conservative management with (A) all‐cause mortality; (B) hospitalization for cardiovascular causes; (C) stroke; and (D) cardiac‐specific mortality. AVR indicates aortic valve replacement; CM, conservative management; and RR, risk ratio.

All included trials reported data on all‐cause mortality, cardiovascular hospitalization, and stroke. Significant reductions were observed among patients receiving early intervention compared with receiving conservative treatment on all‐cause mortality (10.0% versus 13.7%; RR, 0.74 [95% CI, 0.55–0.99]; I^2^=58%) (Figure 3A), hospitalization for cardiovascular causes (14.6% versus 32.5%; RR, 0.48 [95% CI, 0.39–0.58]; I^2^=26%) (Figure 3B), and stroke (4.5% versus 7.2%; RR, 0.62 [95% CI, 0.40–0.95]; I^2^=0%) (Figure 3C). Three trials (526 patients; early intervention: 264, conservative treatment: 262) reported cardiac‐specific mortality. No significant difference was found between the groups (8.3% versus 16%; RR, 0.68 [95% CI, 0.40–1.16]; I^2^=65%) (Figure 3D). Myocardial infarction was reported in 2 trials with 151 patients in each group, and there was no significant difference between patients assigned to early intervention and conservative treatment (0.6% versus 4.6%; RR, 0.21 [95% CI, 0.04–1.19]; I^2^=0%) (Figure S3). Funnel plots did not reveal obvious publication bias for the outcomes (Figures S4 through S8).

## DISCUSSION

The meta‐analysis of RCTs compared an early interventional strategy with a conservative strategy in asymptomatic patients with severe AS. The analysis demonstrated the superiority of early intervention over CM with respect to the primary composite outcome of all‐cause mortality, hospitalization for cardiovascular causes, stroke, and myocardial infarction, and with respect to the all‐cause mortality, hospitalization for cardiovascular causes, and stroke, respectively. However, significant differences in the rates of cardiac‐specific mortality and myocardial infarction were not apparent.

For patients with asymptomatic severe AS, the decision to pursue early AVR or adopt a conservative strategy until symptoms develop remains a matter of debate.^24^, ^25^ Traditionally, a watchful waiting approach has been preferred. However, accumulating evidence indicates that asymptomatic severe AS often progresses and leads to serious adverse outcomes if left untreated. An observational study by Pellikka et al., following 622 asymptomatic patients with severe AS, found the proportion of freedom from AVR or death was 80%, 63%, and 25% at 1, 2, and 5 years, respectively.^26^ Another large cohort study by Lancelotti et al. pooled data from 10 clinics across Europe, the United States, and Canada.^27^ Eight hundred sixty‐one asymptomatic patients with severe AS were included in this cohort. The proportion of freedom from death was 93%, 86%, and 75% at 2, 4, and 8 years, respectively, while AVR‐free survival was 54% and 32% at 2 and 4 years, respectively. Several meta‐analyses have compared the early SAVR or TAVR with CM among asymptomatic patients with severe AS, suggesting that early surgical interventions were associated with a lower rate of all‐cause mortality, cardiac‐specific mortality, noncardiac mortality, and heart failure hospitalization compared with CM. However, most included studies were nonrandomized and observational, with several having small sample size (<100) or lacking the baseline information of participants,^12^, ^13^, ^28^ making them susceptible to bias due to unmeasured confounding variables or systematic differences between early interventions group and conservative treatments groups.

In our analysis, although early intervention strategies outperformed CM in reducing the composite outcome, no significant differences were observed in the rates of cardiac‐specific mortality, which was inconsistent with previous meta‐analysis. Actually, among the RCTs in our analysis, only the RECOVERY trial reported significant differences between early intervention and conservative treatment strategies with respect to the all‐cause and cardiac‐specific mortality. This discrepancy may primarily be attributed to selection bias within the included trials. Participants in the RECOVERY trial had more serious AS than other trials, with a mean aortic valve area of 0.63 m^2^, a transaortic pressure gradient of 64 mm Hg, and an aortic jet velocity of 5.14 m/s. The severity of stenosis may influence the outcomes of intervention. A large multicenter study by Miyake et al. found no difference in survival or cardiovascular death–free survival between the initial AVR and conservative groups; however, subgroup analysis showed better outcomes in patients with a peak aortic jet velocity ≥4.5 m/s undergoing initial AVR.^29^ Lancellotti et al. suggested that a peak aortic jet velocity >5 m/s was associated with increased all‐cause and cardiac‐specific mortality in the absence of AVR.^27^ Additionally, participants in the RECOVERY trial had a younger average age and lower rates of comorbidities, including high body mass index, hypertension, diabetes, hyperlipidemia, and coronary artery disease, which were associated with a lower rate of early procedural risks. The current trial suggests that findings of improved mortality with early intervention may not be generalizable to the broader, older population with asymptomatic severe AS and a higher burden of comorbidities. In fact, the mean younger age in the early AVR group compared with the CM group of several observation studies may, in part, have driven the ability of previous meta‐analyses to show such a significant difference in all‐cause mortality and cardiac‐specific mortality.^14^, ^15^, ^30^, ^31^ The outcome of our meta‐regression analysis also suggests that part of the observed heterogeneity may be attributable to differences in baseline age and severity of AS across studies. Follow‐up durations may be another potential factor contributing to the lack of significant differences. The RECOVERY trial featured a longer clinical follow‐up compared with the other trials. A substantially higher proportion of patients in the conservative treatment arm of the AVATAR trial remained AVR‐free during follow‐up compared with those in the RECOVERY trial. Additionally, in the AVATAR trial, although no significant differences in all‐cause mortality or cardiac‐specific mortality were observed between the 2 groups during the initial short‐term follow‐up, prolonged follow‐up revealed a significantly lower all‐cause mortality rate in the early‐intervention group. Variations in disease progression and intervention strategies during follow‐up may have contributed to the heterogeneity in all‐cause and cardiac‐specific mortality outcomes.

Considering the impact of early AVR on symptom reduction and prevention of hospitalizations in older populations is also crucial. The current analysis demonstrated significant reductions in hospitalization for cardiovascular causes and stroke among patients undergoing early aortic valve intervention, which is consistent with previous analysis. Asymptomatic severe AS is associated with the progression of irreversible cardiac damage, which cannot be reversed by medication or later AVR and ultimately leads to severe symptoms or adverse outcomes.^32^ Early interventions may thus prevent the irreversible cardiac damage caused by progressive valve obstruction and reduce the risk of adverse outcomes. Furthermore, because the trials were open‐label, patients undergoing early interventions may have received more comprehensive follow‐up and perioperative monitoring, leading to earlier and more timely management to minimize adverse outcomes such as heart failure, stroke, or myocardial infarction.

According to the 2021 ESC/EACTS (European Society of Cardiology (ESC) and European Association for Cardio‐Thoracic Surgery) guidelines, aortic valve replacement is a class I recommendation for symptomatic patients with severe AS, as well as for asymptomatic patients who have a left ventricular ejection fraction of <50%, a positive result on stress testing, or other clinical indications that necessitate surgical intervention.^7^ Our findings suggest that early intervention may be necessary in asymptomatic patients with severe AS to prevent the onset of symptoms and reduce the risk of unplanned hospitalizations. Nonetheless, according to our analysis, it is essential to take into account not only the degree of aortic valve stenosis but also patient‐specific factors, including age, the presence of comorbidities, and overall clinical status in shared decision‐making. Additionally, given the small number of included studies and the potential instability in estimating between‐study variance, we adopted a fixed‐effect model interpreted as a particular weighted average of the included studies, rather than assuming a common underlying effect.^20^, ^21^ While this approach is statistically appropriate under these conditions, it limits the generalizability of our findings, because the estimated effect reflects only the specific studies analyzed.^21^ Furthermore, the wide PI observed suggests that, despite the pooled RR indicating a beneficial effect (RR <1), the true effect in some settings may be null or even adverse. This highlights the importance of study context, design, and population differences in interpreting summary estimates, particularly in meta‐analyses with few studies. These findings support the need for further high‐quality, adequately powered studies to confirm the robustness and external validity of the observed treatment effect.

The meta‐analysis of randomized controlled trials supported early intervention with SAVR or TAVR in asymptomatic severe AS. However, the results should be interpreted with caution in clinical practice due to several potential limitations. First, the mean age of participants ranged from 65 to 76 years in our analysis, whereas AS is more prevalent in the elderly population, affecting 4% of individuals >70 years old and 10% of those >80 years old.^33^ Future studies should focus on the elderly populations. Second, SAVR and TAVR were both considered herein. Despite a previous RCT with a small sample size reporting that low‐risk asymptomatic patients with severe AS who underwent TAVI had clinical outcomes comparable to those of SAVR, the effects of these 2 different interventions warrant consideration in clinical practice.^34^ Third, all the RCTs included were not blinded because the interventions were clearly visible. The open‐label design might have contributed to the observed benefits of the early interventions. Fourth, there was a lack of standardization in the definitions of events across the included trials. Only the AVATAR trial reported specific definitions and the use of an adjudication committee for outcome verification, whereas such information was unavailable in other studies. This lack of standardization may have introduced heterogeneity and should be considered when interpreting our results. Additionally, in EVOLVED and EARLY TAVR, the percentage of female participants was only 30%, which may increase the risk of bias.

## CONCLUSIONS

Our meta‐analysis of RCTs demonstrated that, in patients with asymptomatic severe AS, early aortic valve interventions outperformed CM in reducing the composite outcome of all‐cause mortality, cardiovascular hospitalization, stroke, and myocardial infarction. However, significant differences in the rates of cardiac‐specific mortality were not apparent.

## Sources of Funding

This work was financially supported by Hunan Provincial Health High‐Level Talent Scientific Research Project (R2023017 to CF), the National Natural Science Foundation of China (82200323, 82470302 to CF).

## Disclosures

None.

## Supporting information

Tables S1–S4
Figures S1–S8
