# Supplementary material for: Early Aortic Valve Replacement of Asymptomatic Severe Aortic Stenosis: A Meta‐Analysis of Randomized Controlled Trials
Source: J Am Heart Assoc. 2025 Aug 20;14(16):e041283. doi: 10.1161/JAHA.125.041283 (PMC12533610; doi:10.1161/JAHA.125.041283)
Supplement: Supplementary file 1 — Tables S1–S4 Figures S1–S8 [file JAH3-14-e041283-s001.pdf]

Supplemental Materials

Table S1. The search details

Table S1a. Search strategy with PubMed

**PubMed** (Date of most recent search, December 4, 2024)		
**Number**	**Searches**	**Results**
#1	"aortic valve replacement"[Title/Abstract] OR "intervention"[Title/Abstract] OR "transcatheter aortic valve replacement"[Title/Abstract] OR "TAVR"[Title/Abstract] OR "TAVI"[Title/Abstract] OR "SAVR"[Title/Abstract] OR "transcatheter aortic valve implantation"[Title/Abstract]	936972
#2	"aortic stenosis"[Title/Abstract] OR (("aorta"[MeSH Terms] OR "aorta"[All Fields] OR "aortas"[All Fields] OR "aorta s"[All Fields] OR "aortae"[All Fields]) AND "valve stenosis"[Title/Abstract]) OR "aortic valve stenosis"[Title/Abstract] 28385	371721
#3	asymptomatic"[Title/Abstract]	201058
#4	#1 and #2 and #3	627

Table S1b. Search strategy with Web of Science

**Web of Science** (Date of most recent search, December 4, 2024)		
**Number**	**Searches**	**Results**
#1	((TS= (aortic stenosis)) OR TS= (aorta valve stenosis)) OR TS= (aortic valve stenosis)	77917
#2	TS=(asymptomatic)	258304
#3	(((((TS=(aortic valve replacement)) OR TS=(intervention)) OR TS=(transcatheter aortic valve replacement)) OR TS=(TAVR)) OR TS=(TAVI)) OR TS=(SAVR) OR TS=(transcatheter aortic valve implantation)	2709257
#4	#1 AND #2 AND #3	1438

Table S1c. Search strategy with Embase

**Embase** (Date of most recent search, December 4, 2024)		
**Number**	**Searches**	**Results**
#1	'aortic stenosis':ab,ti OR 'aorta valve stenosis':ab,ti OR 'aortic valve stenosis':ab,ti	42519
#2	asymptomatic:ab,ti	294014
#3	'aortic valve replacement':ab,ti OR 'intervention':ab,ti OR 'transcatheter aortic valve replacement':ab,ti OR 'tavr':ab,ti OR 'tavi':ab,ti OR 'savr':ab,ti OR 'transcatheter aortic valve implantation':ab,ti	1292150
#4	#1 AND #2 AND #3	1095

Table S1d. Search strategy with Cochrane Library

**Cochrane Library** (Date of most recent search, December 4, 2024)		
**Number**	**Searches**	**Results**
#1	(aortic stenosis):ti,ab,kw OR (aorta valve stenosis):ti,ab,kw OR (aortic valve stenosis):ti,ab,kw	2459
#2	(asymptomatic):ti,ab,kw	15352
#3	(aortic valve replacement):ti,ab,kw OR (intervention):ti,ab,kw OR (transcatheter aortic valve replacement):ti,ab,kw OR (TAVR):ti, ab,kw OR (TAVl):ti, ab,kw OR (SAVR):ti,ab,kw OR (transcatheter aortic valve implantation):ti,ab,kw	655340
#3	#1 AND #2	97

Table S2. Overview of the GRADE approach

Certainty assessment							No of patients		Effect			
**No of studies**	**Study design**	**Risk of bias**	**Inconsistency**	**Indirectness**	**Imprecision**	**Other considerations**	**Early AVR**	**CM**	**Relative (95% CI)**	**Absolute (95% CI)**	**Certainty**	**Importance**
**Composite outcome of all-cause mortality, hospitalization for cardiovascular causes, and stroke and myocardial infarction.**												
4	RCTs	serious[a]	serious[b]	not serious	not serious	none	210/719 (29.2%)	380/708 (53.7%)	**RR 0.51** (0.38 to 68.00)	**263 fewer per 1,000** (from 333 fewer to 1,000 more)	⊕⊕◯◯ Low[a,b]	CRITICAL
**All-cause mortality**												
4	RCTs	serious[a]	not serious	not serious	not serious	none	72/719 (10.0%)	91/708 (12.9%)	**RR 0.75** (0.50 to 1.14)	**32 fewer per 1,000** (from 64 fewer to 18 more)	⊕⊕⊕◯ Moderate[a]	CRITICAL
**Hospitalization for cardiovascular causes**												
4	RCTs	serious[a]	not serious	not serious	not serious	strong association[c]	105/719 (14.6%)	230/708 (32.5%)	**RR 0.35** (0.20 to 0.64)	**211 fewer per 1,000** (from 260 fewer to 117 fewer)	⊕⊕⊕⊕ High[a]	CRITICAL
**Stroke**												
4	RCTs	serious[a]	not serious	not serious	not serious	none	32/719 (4.5%)	51/708 (7.2%)	**RR 0.62** (0.40 to 0.95)	**27 fewer per 1,000** (from 43 fewer to 4 fewer)	⊕⊕⊕◯ Moderate[a]	CRITICAL
**Cardiovascular mortality**												
3	RCTs	serious[a]	serious[b]	not serious	not serious	none	22/264 (8.3%)	42/262 (16.0%)	**RR 0.50** (0.15 to 1.63)	**80 fewer per 1,000** (from 136 fewer to 101 more)	⊕⊕◯◯ Low[a,b]	CRITICAL

a, open-label design of the RCTs; **b,** high heterogeneity with I^2 >50%; **c,** large effect with RR<0.50.
AVR, aortic-valve replacement; **CM,** conservative management; **CI,** confidence interval; **RR,** risk ratio; **RCT,** randomized controlled trials.

Table S3. Sensitivity analysis with the leave-one-out method for the primary efficacy outcome

Study Omitted	RR (95% CI)	I^2
RECOVERY, 2020	0.57 [0.50, 0.65]	0%
AVATAR, 2021	0.51 [0.35, 0.76]	68%
EVOLVED, 2024	0.44 [0.29, 0.68]	71%
EARLY TAVR, 2024	0.44 [0.26, 0.73]	70%

RR, Risk ratio; CI, confidence interval.

Table S4. Meta-regression analysis of factors affecting heterogeneity in primary composite outcome

Moderator	Coefficient(β)	SE	Z-value	*P*-value
**Age**	**0.0533**	**0.0232**	**2.3003**	**0.0214**
BMI	0.2332	0.126	1.843	0.0654
**Peak aortic jet velocity**	**-1.1208**	**0.5501**	**-2.0375**	**0.0416**
Follow up duration	-0.0194	0.0153	-1.2716	0.2035

SE, standard error

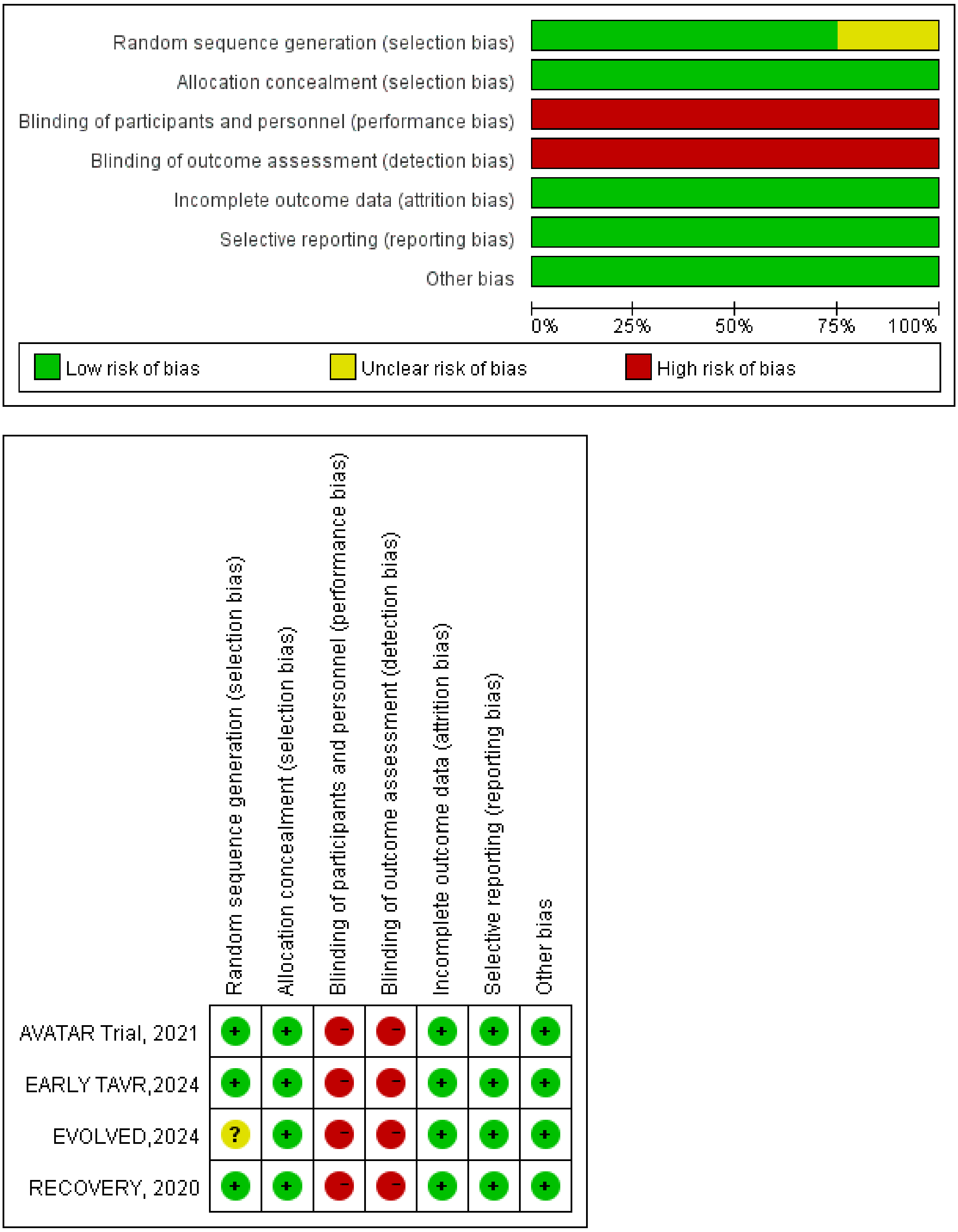

Figure S1. Risk of bias assessment for included studies

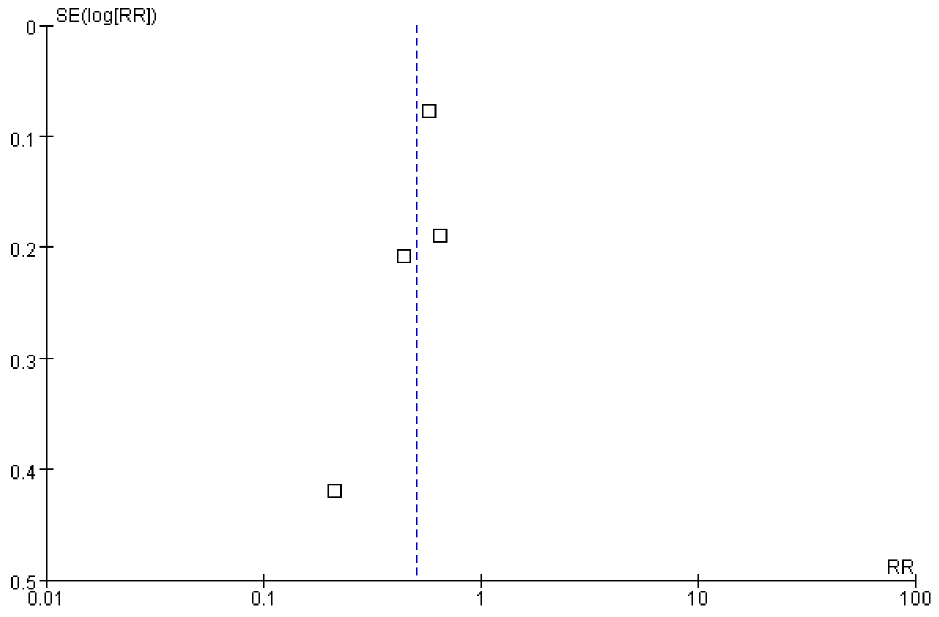

Figure S2. Funnel plots illustrating the publication bias on the composite outcome of all-cause mortality, hospitalization for cardiovascular causes, stroke and myocardial infarction.

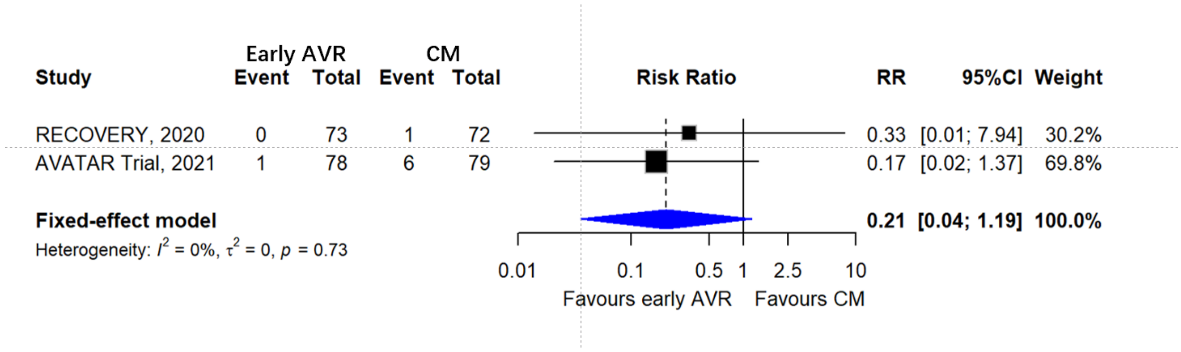

Figure S3. Forest plot illustrating the association of early AVR and conservative management with myocardial infarction. AVR, aortic valve replacement; CM, conservative management; CI, confidence interval.

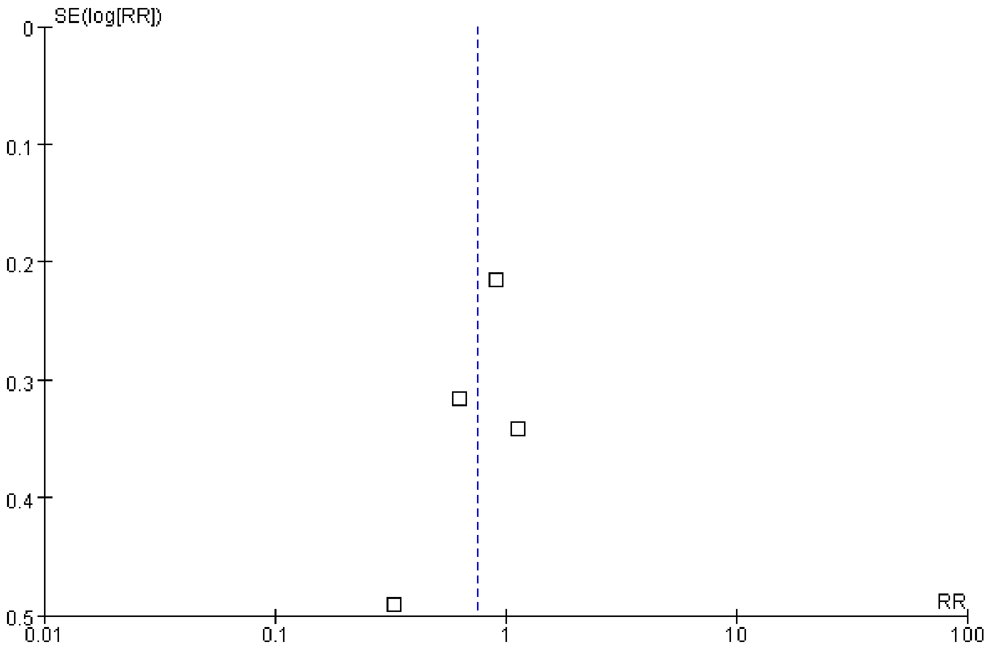

Figure S4. Funnel plots illustrating the publication bias on all-cause mortality.

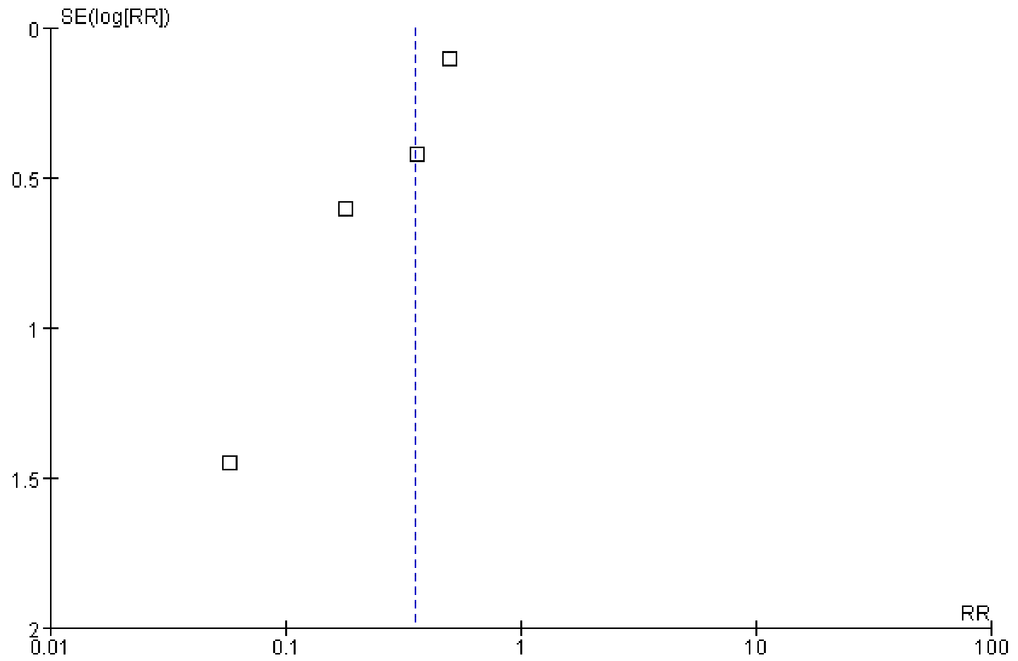

Figure S5. Funnel plots illustrating the publication bias on hospitalization for cardiovascular causes.

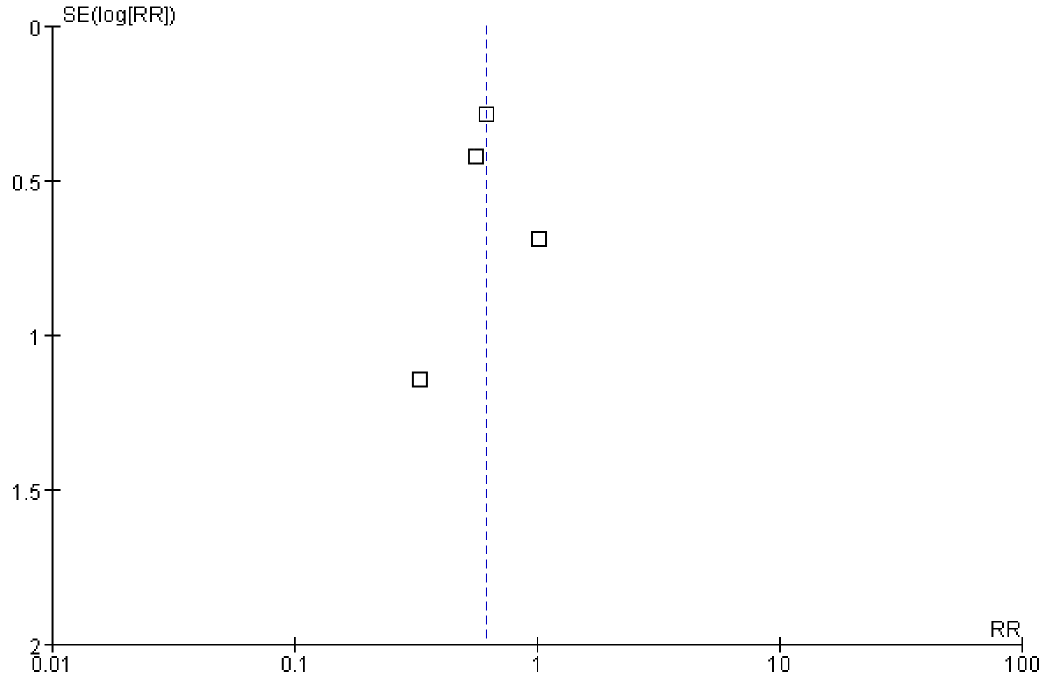

Figure S6. Funnel plots illustrating the publication bias on stroke.

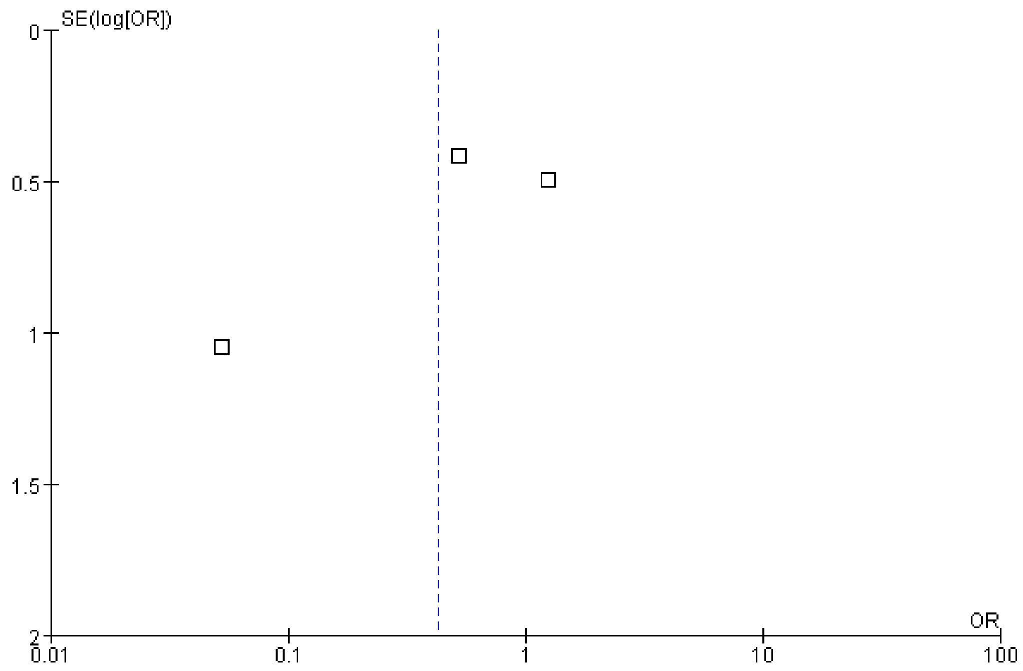

Figure S7. Funnel plots illustrating the publication bias on cardiovascular mortality.

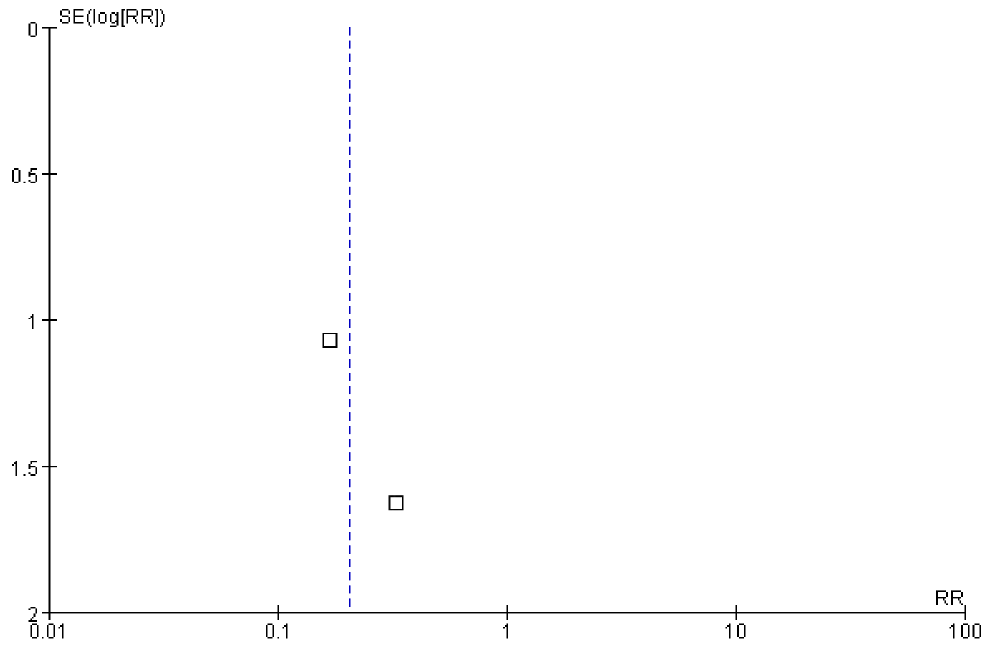

Figure S8. Funnel plots illustrating the publication bias on myocardial infarction.
